# Sigmoid Carcinoma in an Inguinal Hernia: A Blessing in Disguise?

**DOI:** 10.1155/2013/314394

**Published:** 2013-12-05

**Authors:** P. B. Salemans, G. F. Vles, S. A. F. Fransen, R. M. Smeenk

**Affiliations:** Atrium Medical Centre, Department of General Surgery, Henri Dunantstraat 5, 6419 PC Heerlen, The Netherlands

## Abstract

Colorectal cancer is a rising problem, as the incidence increases with age. In most cases the goal of treatment is oncological resection followed by adjuvant chemotherapy in order to optimize the survival. In this case report we present a 93-year-old patient with a sigmoid carcinoma inside an irreducible inguinal hernia, which was diagnosed prior to surgery. We chose to perform a sigmoid resection through an oblique inguinal incision as a safer alternative to laparotomy.

## 1. Introduction

Colorectal cancer (CRC) is the third most commonly diagnosed cancer in males and the second in females. It is estimated that over 1.2 million new cases and 608.700 deaths have occurred in 2008 [1].

In most cases of CRC, the primary goal is curative treatment. This means a radical resection and, if necessary, adjuvant chemotherapy.

In CRC, as the incidence increases with age, there is a peak of 350 cases/100.000 individuals above the age of 80 [2]. This ever-increasing group is especially at risk for complications after colorectal surgery, as a result of diminished overall health, functional decline, and limited data to guide decisions. The overall relative survival rate after colorectal surgery (acute and elective) at 2 years in this group of patients is 0.62 compared to the under 65-year group. Postoperative complications like pneumonia, cardiovascular events, and cerebrovascular accidents, as well as the length of hospital stay, are significantly increased with advancing age [3, 4].

Initial common symptoms in patients presenting with colon cancer are abdominal pain, change in bowel habits, melaena, and general weakness [5]. Occasionally a patient presents with what we consider a blessing in disguise. Less than 1 out of 200 cases of CRC is localized within an inguinal hernia [6]. In most instances, the sigmoid colon is herniated through the left inguinal canal [7]. It is often long-standing and does not cause symptoms. Two recent reviews of the literature demonstrated that carcinoma in an inguinal sac occurs almost exclusively in elderly men, mostly originates from the sigmoid colon, and becomes incarcerated in the left groin [8, 9].

We present a 93-year-old man with a sigmoid carcinoma in a left-side irreducible inguinal hernia, which was diagnosed prior to surgery and was resected through a standard oblique inguinal incision.

## 2. Case Report

**Figure 1:**
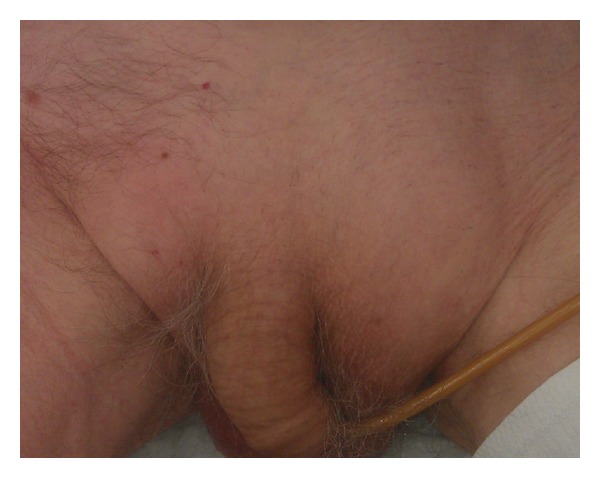
An irreducible, nontender mass in the left groin.

**Figure 2:**
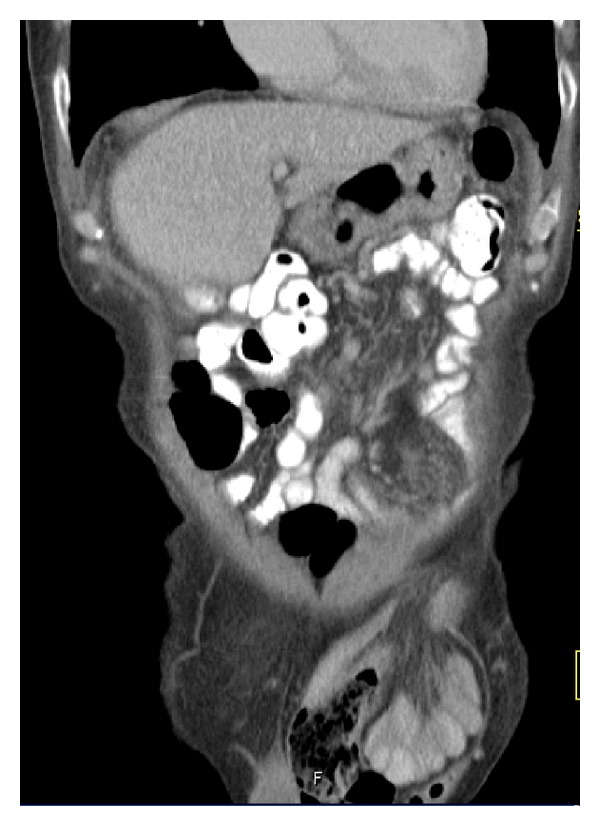
Coronal CT showing herniation of colon and mesentery through the inguinal canal.

A 93-year-old man with a medical history of hypertension, left bundle branch block, and macular degeneration was admitted to the emergency department because of rectal bleeding and abdominal pain. Physical examination revealed a nontender, irreducible mass in the left groin (Figure 1). Rectal palpation was normal. Relevant blood testing results were haemoglobin 6.8 mmol/L and carcinoembryonic antigen 1.9 *μ*g/L. An abdominal computed tomography (CT) scan revealed an inguinal hernia-containing tumour (Figure 2). A colonoscopy was performed and showed an obstructing tumour in the sigmoid colon. A biopsy was taken and the tumour was spotted. Histological examination confirmed adenocarcinoma.

**Figure 3:**
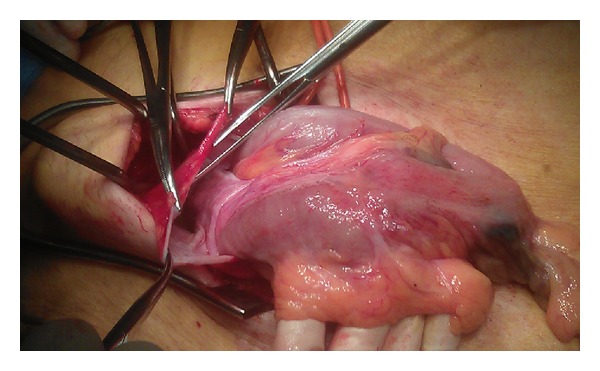
Opening of the inguinal hernia sac revealing the sigmoid colon with spotted tumour.

**Figure 4:**
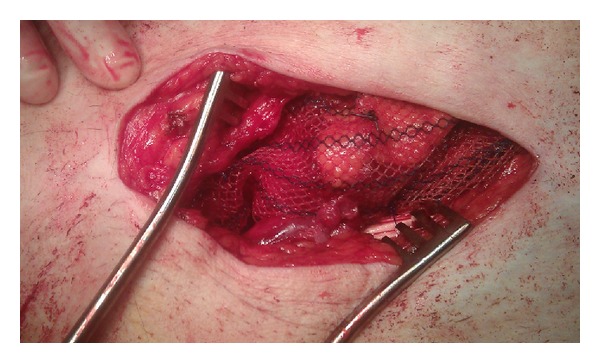
Restoration of the abdominal wall using a mesh according to Lichtenstein.

Considering age, vitality, and associated peroperative risks of this patient, our team of gastrointestinal surgeons held the opinion that a low anterior resection would be too much of a burden and it was decided to perform a tumour resection through a standard oblique inguinal incision. Peroperative findings were a large left-sided inguinal hernia, which contained the sigmoid colon with the spotted tumour (Figure 3). Resection of the sigmoid colon and mesentery, with primary anastomoses, was performed. The remaining sigmoid colon was reduced in the peritoneal cavity and the abdominal wall was restored according to Lichtenstein (Figure 4) [10]. The patient was discharged in good clinical condition 7 days after surgery.

Histological examination of the specimen revealed a radically resected, moderately differentiated adenocarcinoma with invasion in the subserosal fat. None of 9 lymph nodes was tumour positive. No adjuvant treatment was given.

## 3. Discussion

In the case reported above, a sigmoid resection was performed through the inguinal canal in order to avoid a laparotomy, minimizing surgical risks. Unfortunately, the performed resection could not be considered optimal from an oncological point of view, as the lymph node yield was less than 12. Although it minimizes the surgical risk during operation by avoiding a laparotomy, one can wonder if this risk reduction has benefits over a suboptimal oncological treatment. For example, Steele et al. [7] found an association between low lymph node yield and decreased overall survival. They suggest that, regardless of age, a proper oncological resection with at least 12 lymph nodes should be conducted during surgery. On the contrary, they also found a negative correlation between advancing age and lymph node yield in nonmetastatic colon cancer, suggesting that in the oldest of the old a fully satisfying specimen cannot always be obtained.

In stage II or stage III colon cancer, the survival rates in patients receiving adjuvant chemotherapy can be up to 16% compared to patients without adjuvant chemotherapy [8, 9]. The elderly also benefit from adjuvant chemotherapy, without a significant increase in toxicity compared to younger patients [11]. However, in people over 85 years of age there is no consensus or supportive evidence whether this group should also receive adjuvant chemotherapy. There is also no evidence to support a certain approach in elective surgery for the inguinal carcinomas of the colon. In most cases, described surgery was performed in an emergency setting or carcinomas were found by surprise, resulting in suboptimal treatment. Often an extra abdominal incision was necessary to perform resection, which increased the risks of complications and mortality [12–16].

In conclusion, we resected a sigmoid carcinoma in a hernia sac through a standard oblique incision in a 93-year-old patient. Therefore surgical risks were minimized and the patient could be discharged relatively short after operation. Although the specimen was oncologically not fully satisfying, we consider the current evidence too small to justify more invasive surgery in order to achieve a potentially oncologically better resection in this group of vulnerable patients.

In our opinion, a resection of a sigmoid carcinoma in an irreducible inguinal hernia through an oblique inguinal incision can be an attractive alternative for the very old patients.
